# Food Environment around Schools: A Systematic Scope Review

**DOI:** 10.3390/nu14235090

**Published:** 2022-11-30

**Authors:** Fabiana Chagas Oliveira de França, Iziane da Silva Andrade, Renata Puppin Zandonadi, Karin Eleonora Sávio, Rita de Cassia Coelho de Almeida Akutsu

**Affiliations:** 1 Nutrition School, Federal University of Bahia-Augusto Viana, s/n-Palácio da Reitoria, Canela, Salvador 40110-907, BA, Brazil; 2 Health Sciences Center, Federal University of Reconcavo da Bahia-Rua Rui Barbosa, 710-Centro, Cruz das Almas 44574-490, BA, Brazil; 3 Department of Nutrition, University of Brasilia, Brasilia 70910-900, DF, Brazil

**Keywords:** schools, students, food environment, foodservice, systematic review

## Abstract

The present systematic scope review intended to compile state-of-the-art information about the food environment around schools, exploring the main methods used to describe the food environment around schools as well as the possible effects that this environment can promote on the health of children and adolescents. The preferred reporting items for systematic reviews and meta-analyses—extension for scoping reviews (PRISMA-ScR) checklist and guidelines were followed to ensure a robust and repeatable methodological process. A systematic search was performed in the following electronic databases: MEDLINE, Embase, Science Direct, Web of Science, LILACS, and Scopus, as well as in related articles, a manual search of reference lists and gray literature. Forty-six studies were selected. There was no standardization regarding distances from food establishments to schools, methods of analysis, and software used. The food environment around the schools was characterized by the wide availability of food establishments, especially fast food, convenience stores, supermarkets, and grocery stores known for offering a wide variety of unhealthy foods. Regarding the correlations with the health of children and adolescents, the evidence points to possible interferences of the food environment known as obesogenic, but it cannot be related only to the school environment since most of the acquisition and consumption of food usually happens around family homes. Conducting standardized and comprehensive studies evaluating food choices in the school environment and their interrelationships is very important to ensure children’s food and nutrition security and minimize negative health outcomes in the medium and long term.

## 1. Introduction

The school environment plays an essential role in students’ dietary patterns. Studies have shown that the variety and quality of food available around schools can influence this population’s health and nutrition outcomes [1,2,3]. When discussing the school food environment, there are several studies to unravel how and why these individuals make their food choices, as well as the impact that these choices have throughout their adult life and senescence [4,5,6,7].

The food environment includes physical, economic, political, and sociocultural aspects, opportunities, and conditions that influence people’s food and beverage choices and nutritional status [8,9]. Characteristics of the school food environment, such as targeted marketing, availability, and access to unhealthy foods, combined with a sedentary lifestyle, contribute to the obesogenic school environment, which influences the nutritional status of children and adolescents [10,11,12,13]. The obesogenic environment concerns the environmental conditions that influence individuals and populations to choose lifestyles that promote obesity, involving the availability of unhealthy foods around individuals and lack of physical activity [10,11,12,14]. Epigenetic studies point to the effects of a child’s diet rich in fats and simple sugars in increasing the probability of occurrence of chronic non-communicable diseases, such as obesity, in the medium and long term [15,16].

Schools are generally surrounded by canteens, grocery stores, restaurants, fast food, and snack bars. This can determine the type of food that this target audience consumes, limiting the offer of healthy possibilities of choice, either by availability, or the portion size, due to the high cost, among other factors. In addition, it is possible to verify in many places the presence of street vendors that present a variety of snacks containing high levels of fats and sugars. When consumed in excess, these foods can contribute to the non-guarantee of food and nutrition security (FNS) to students. In this context, the proximity of schools to establishments that sell food can be a potentiating or protective factor for obesity, depending on the availability, access, and types of food sold in these places, as well as the students’ access to food education and nutrition, so that they can get to know foods better and have more autonomy to make healthy choices to compose their eating routine.

**Figure 1:**
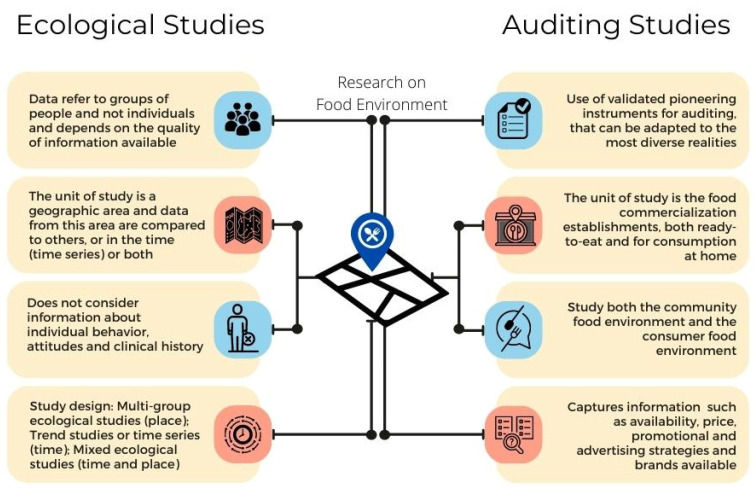
Characteristics of ecological studies and audit studies to describe the food environment.

The study of food environments around schools presents at least two forms of investigation: ecological studies and auditing studies. Ecological studies determine the food environment from secondary data from the registration of establishments that sell food, with an observation regarding the qualitative aspects of the food served in these environments (restaurants, cafeterias, canteens, grocery stores) (Figure 1) [2,3]. Auditing studies aim to collect information on the quality and availability of food and consumer products within food vendors’ establishments (Figure 1) [17]. The methodologies are not exclusive and have been combined in some studies to validate instruments [18,19,20,21].

Research on the food environment using ecological studies has developed in recent decades in high-income countries, especially North America, in response to the high prevalence of overweight, obesity, and chronic non-communicable diseases [22,23,24,25]. However, despite studies proving the importance of food and obesogenic environments in childhood and adolescence [11,26,27,28], little is known about the state of science and the emerging body of evidence from the school setting, where children and adolescents spend most of their day. This is a significant research gap, given the susceptibility of the target audience to the supply of (often unhealthy) foods, the eating habits that are strengthened from this daily exposure and consumption, and the challenge related to nutrition and public health to revert potentially worrying situations in this regard.

Therefore, this systematic scoping review aims to fill this gap, addressing the following questions concerning the food environment around schools: (1) What are the characteristics of the schools where the food environment was evaluated? (2) What types of food establishments were investigated when studying the food environment around schools? (3) What methodologies were used to assess food establishments’ density and/or proximity to schools? (4) What are the main findings on the associations between exposure to the food environment near schools and dietary, nutritional, and health outcomes? The synthesis of the knowledge of this review is intended to compile state-of-the-art information about the food environment around schools, strengthening the need for a careful look at this public environment, and encouraging the adoption of measures that can guarantee food and nutritional security for this population and reduce future risks and harms.

## 2. Materials and Methods

A systematic scoping review was performed as a salient approach possibility when mapping the existing literature in a given field [29,30]. The preferred reporting items for systematic reviews and meta-analyses—extension for scoping reviews (PRISMA-ScR) checklist and guidelines were followed to ensure a robust and repeatable methodological process [30].

### 2.1. Search Strategy

A systematic search was performed in the following electronic databases: MEDLINE, Embase, Science Direct, Web of Science, LILACS, and Scopus, as well as in related articles, a manual search of reference lists and gray literature. There were no language and time restrictions. Keywords were “schools”, “students”, “adolescents”, “fast food”, “food environment”, “restaurants”, equivalent Descriptors in Health Sciences/Medical Subject Headings (DeCS/MeSH) descriptors, and Boolean operators AND and OR.

### 2.2. Eligibility Criteria

For the inclusion of studies, the following were considered: (1) studies carried out with children and adolescents (< 19 years old), (2) studies carried out in pre-schools and schools, and (3) studies related to the food environment. Exclusion criteria were: scoping reviews, systematic reviews and meta-analyses, letters, editorials, and abstracts/articles repeating information from a previously included population.

### 2.3. Studies Selection and Data Collection

We conducted the selection in two phases. In the first phase, two reviewers (F.C.O.F. and I.S.A.) independently reviewed the titles and abstracts of all manuscripts identified from databases. The reviewers discarded the articles that did not meet the eligibility criteria. In the second phase, the reviewers applied the eligibility criteria to the full texts of the selected articles. In the two phases, in cases of disagreement, the issue was discussed until a consensus was obtained. In cases where there was no consensus, the third reviewer made the final decision (R.C.C.A.). The complete text of the manuscripts was considered for the final selection. F.C.O.F. and I.S.A., the reviewers, critically evaluated the list of references of the selected studies. After this phase, two reviewers (F.C.O.F. and I.S.A.) extracted data from the manuscripts. The third examiner (R.C.C.A.) and expert (R.P.Z.) added additional studies.

The Mendeley reference manager software was used for reference management, first for screening titles and abstracts and later for grouping the selected complete works. When the study met the inclusion and exclusion criteria, data extraction was performed independently and in duplicate by two researchers, including authors, year, and country in which the study was performed, sample size, unit of analysis, number and types of establishment, number and types of school, number of students served, characteristics of the food environment, and possible health outcomes explained. The search started in January 2022, and the final database search occurred in June 2022.

The risk of bias was independently assessed by the reviewers (F.C.O.F. and I.S.A.), using the meta-analysis of statistics assessment and review instrument (MAStARI) developed by the Joana Briggs Institute [31]. A checklist containing the following questions was applied: (1) Were the criteria for inclusion in the sample clearly defined? (2) Were the study subjects and the setting described in detail? (3) Was the exposure measured in a valid and reliable way? (4) Were objective, standard criteria used to measure the condition? (5) Were confounding factors identified? (6) Were strategies to deal with confounding factors stated? (7) Were the outcomes measured in a valid and reliable way? (8) Was appropriate statistical analysis used?

For these questions, the possible answers were “yes”, “unclear”, “no”, or “not applicable”. The risk of bias was considered high if the study reported “yes” score of up to 49%; moderate between 50% and 69%; and low of more than 70%.

## 3. Results and Discussion

**Figure 2:**
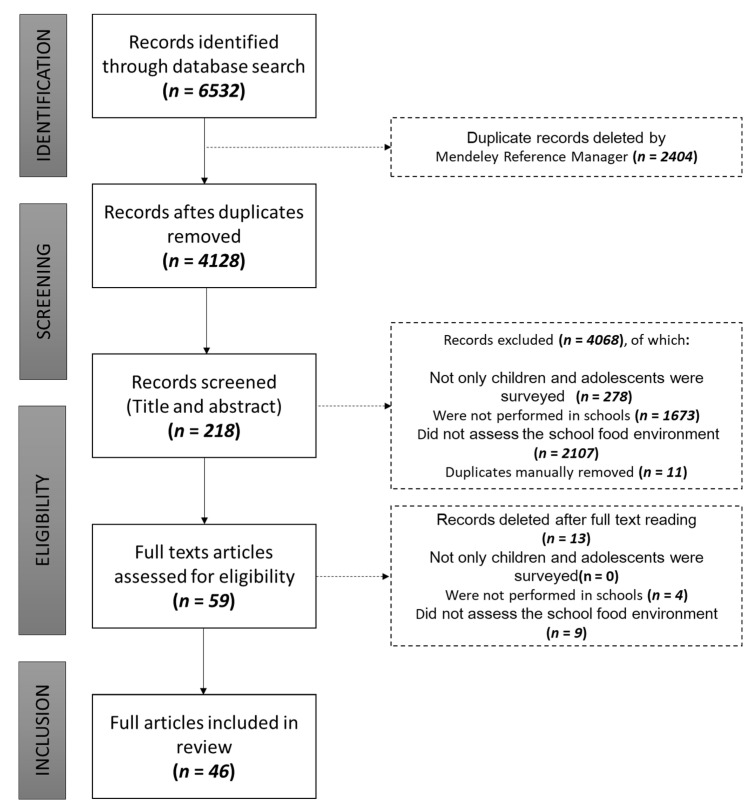
Evidence sources selection flowchart according to PRISMA-ScR [23].

A total of 6532 articles were found by searching the databases. After analysis of duplicates and selection by title and abstract, 6314 were excluded, and 218 articles with data of interest were identified. Of these, 159 were still excluded because they did not meet the eligibility criteria, and 59 citations were selected for a complete reading. After reading the articles in full, 46 studies carried out in pre-schools or schools that brought data on the food environment inside and/or around the schools were maintained. The study selection flowchart was constructed as indicated by PRISMA-ScR (Figure 2).

The first publication occurred in 2005 and about 60% (*n* = 27) were published between 2010 and 2014. Most studies were performed in North America (*n* = 29), but there were also studies in South America (*n* = 9), Oceania (*n* = 4), and Europe (*n* = 3). A multicenter research paper performed in the United States, Scotland, and Canada was also included. The sample size varied widely from 3 to 31,622 schools analyzed and an equally variable number of students, reaching more than 500,000.

Regarding the overall risk of bias, 78.26% of studies were considered at moderate risk (*n* = 36), 10.87 (*n* = 5) at high risk, and 10.87% (*n* = 5) at low risk (Table 1).

Information on the studies, sample characteristics (size and composition), density analysis units, and/or proximity to food trading establishments, and the types of establishments researched by the authors are presented in Table 2.

### 3.1. Characteristics of the Schools Studied

A total of 31% (*n* = 18) of the studies were conducted only in public schools; 20% (*n* = 9) in both public and private schools, and the others did not classify schools as public or private. About 41% (*n* = 19) of the studies did not specify the school level of education. In the other studies, the schools were classified as pre-schools (*n* = 6), elementary schools (*n* = 24), and high schools (*n* = 13).

The categories of food establishments varied among the studies. Most of the studies (*n* = 41) pre-defined the types of food establishments based on the reality of commerce around the schools and the probability of selling foods rich in sugar and fats. The most common types of food establishments were fast-food restaurants (*n* = 35), convenience stores (*n* = 14), supermarkets (*n* = 12), grocery stores (*n* = 12), and restaurants (*n* = 7).

Foods sold in fast-food restaurants and convenience stores are recognized by some characteristics such as umami flavor (frequent in foods rich in monosodium glutamate) and high energy density. These characteristics compromise satiety and uncontrollable appetite, favoring involuntary and increased consumption of these foods and, consequently, the risk of excess weight. Their commercialization, especially around schools, represents a challenge for promoting healthy eating habits [11,46,68,69,70,71].

Studies reported a greater proximity of food outlets (especially grocery stores and fast-food restaurants) from high schools than preschools or elementary schools. The authors report that the flexibility of schedules, greater independence, and autonomy of high school students can decoy for the food market, in addition to the privileged location of schools on high-traffic roads, coinciding with the location of restaurants and supermarkets [2,13,26,32,35,39,50,64,72].

Public schools (*n* = 9), in general, were more crowded than private schools (*n* = 14). However, private primary schools had greatest exposure to food environment than public schools (which tend to represent low-income groups). Possibly young people perceived as having greater financial means to buy food (especially takeover or fast food) are a more desirable consumer group and are actively targeted.

High schools have 1.61 times more fast-food restaurants and a similar number of convenience stores within walking distance than pre-schools [35,36,39,41,47,73]. Probably because these schools are located in high-traffic urban areas, implying a high potential for selling fast food as a snack option for teenagers. In addition, unlike adolescents, preschool-age children do not yet have the autonomy to walk alone to food outlets and are restricted to products sold at school.

Some authors identified that, compared with schools located in white urban areas or mixed racial/ethnic neighborhoods, schools in urban African American neighborhoods have fewer fast-food restaurants and convenience stores within walking distance [33,35,39,40,41,45,49,56,58,66].

Middle-income neighborhoods have a similar number of fast-food restaurants compared to lower-income neighborhoods. In contrast, higher-income neighborhoods have fewer fast-food restaurants and more restaurants with a wide choice (especially à la carte). Additionally, middle- and upper-income neighborhoods have fewer convenience stores and grocery stores than low-income neighborhoods [33,35,39,40,41,45,49,56,58,66].

Race and low-income data bring us to an important discussion about the availability of healthy foods for marginalized populations, such as mixed racial/ethnic neighborhoods and residents of the urban periphery. Despite not being explicit in the data, it was perceived that the spatial distribution of establishments that sell foods that are considerably cheaper and of low nutritional value, was greater in low-income neighborhoods, which are mostly marginalized due to race/ethnicity and the location of residences. However, in a systematic review article, Mackenbach et al. [9] evaluated studies on the relationship between the school environment, socioeconomic status, and eating behavior and concluded that there was no clear evidence for socioeconomic differences in the association between food environments and dietary behavior. Therefore, more in-depth studies are needed on the access and consumption of foods related to race/ethnicity and income to understand these issues and their connection better.

### 3.2. Availability and Proximity of Food around Schools

The availability of food establishments close to schools was verified in the studies of this review, using a variety of metrics and spatial scales, with the predominant method of characterizing the availability of food in the vicinity of schools from geographic information systems (*n* = 40). The use of geospatial software to delimit a zone around the school or to cover the path between home and school (ranging from 2 to 15 min of walking) was the method most found in studies.

**Figure 3:**
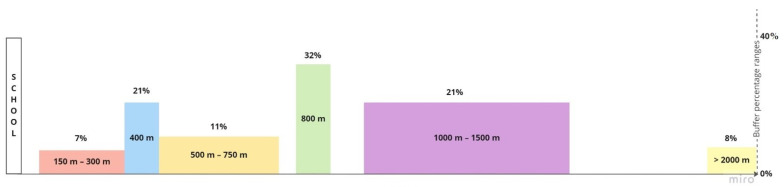
Buffer percentage ranges (*n* = 46).

Based on the information from the studies, it was possible to count the number of food establishments within the specified area (density). Regarding the distance from the school center to the food establishments, the studies ranged from 150 m to 5000 m (Figure 3), with a predominance of analysis of distances of 400 m (*n* = 14) and 800 m (*n* = 21), and between 1000 m and 1500 m (*n* = 14).

Most of the studies using a buffer zone justified the distance used (*n* = 25). Some of them (*n* = 11) mentioned that the distance was established to be consistent with previous studies. Seliske et al. [53] focused on comparing the different lengths to reach an ideal buffer size for analyzing the school environment in Canada, based on students’ possibilities of buying food around schools. The authors considered that the buffer distance of the most suitable road network, when evaluating the food retail environment around Canadian schools, was 1000 m. In agreement with the authors and considering the difficulties in performing our study, it is noteworthy that standardization for the buffer size would be interesting to obtain more consistent data between studies. It could allow comparison among different studies and provide more accurate data to support public policies and initiatives to modify the school food environment.

For studies based on geographic information systems, information on the locations, names, and types of food outlets predominantly came from sizeable secondary data sources, including private companies and local business directories (*n* = 29) or public records, such as census data, tax registration documents, or government food facilities databases (*n* = 8). A minority of authors chose to map the availability of food sold in the surroundings through subjective measures, including questionnaires applied to the school community (*n* = 4) and direct observation by the authors themselves (*n* = 2). Three studies identified food outlets through a questionnaire in which school administrators identified the presence of food outlets’ walkability [46] or a “seven to ten-minute walk from the school” [13,55]. Henriques et al. [66] used an audit tool to record observations of the different types of food outlets found within 500 m of the school.

The inhomogeneity in the choice of distances used for analysis may be due to several factors, such as differences between urban and rural areas, the means of transport used, the distance on foot between the school and the destination, and the distance from commercial food centers. Often, within the urban environment, the options for purchasing food are more diverse and abundant, especially if we consider the distance traveled on foot or the age of the schoolchildren since adolescents have more autonomy to travel and purchase food outside of school.

Using the school as a starting point for purchasing food, the distance to the nearest place is decisive for access and the decision to purchase food. Some authors have noted that the road network and Euclidean distances can produce quite different results in measuring exposure levels [14,32,44,54]. The rectangular street grid that characterizes most cities and the walkable scale make Euclidean measurements less likely to misinterpret as accessibility by schoolchildren who walk across, since the child, in general, will not walk a straight line between two points to travel from the school to the place (needing to follow the path formatted through the streets, generally increasing the route).

When evaluating schools located in rural areas, the authors needed to expand the analysis radius to identify more food establishments [33,40,47,53], implying the possibility of food deserts in those places, despite not being the study objective. Food deserts are often characterized as socioeconomically vulnerable neighbors where individuals have little access to healthy food. Notably, the “desert” component is inherently spatial and relates to the physical lack of food establishments that provide healthy food options in low-income neighborhoods [33,40,47,53].

Seliske et al. [53] conducted a formal analysis to identify which buffer size was the best predictor of eating behaviors and food purchases by analyzing the food retail environment surrounding schools using several buffer sizes. The authors concluded that the 1000 m buffer would be the appropriate size to examine the relationship between the school food retail environment and adolescent eating behaviors. Based on an average walking speed of 4 to 5 km/hour, this distance can be covered in approximately 10 to 15 min. At distances of less than 1000 m, a few schools had at least one food retailer present, suggesting that these buffers were too small to capture a sufficient amount.

Most studies (*n* = 39) identified a significant number of establishments selling unhealthy foods around schools. It can be characterized as food swamps, neighborhoods with many unhealthy food establishments, where robust strategies marketers constantly direct and promote this type of food [56,57,65,67]. As school is the environment where children and adolescents spend most of their day (while they are awake). Considering that in these life stages, there is the formation and consolidation of eating habits [2,11,57,60,61], the high availability of unhealthy foods can promote an inadequate dietary pattern, limit the acceptance of in natura foods, and provide negative health impacts, with a negative outcome in adulthood and senescence [15,16,49].

Curiously, few studies mentioned the existence of a school canteen and did not separate school canteens as the focus of the healthy food trade, although they were included in the food environment. Leite et al. [3] evaluated public and private school canteens and concluded that the presence of a commercial canteen was associated with an increase in the mean attendance score of consumption of ultra-processed foods. Corroborating this study, Rocha et al. [5] concluded that public and private schools that sold soft drinks were associated with higher average consumption of sugar-sweetened beverages among adolescents. The presence of a school canteen (with healthy food) could significantly reduce the attraction to unhealthy foods. However, as they also sell industrialized foods, they can be included in the potential obesogenic environment. The only possibility for them to constitute a protector in relation to food would be to offer only healthy food, as proposed by some national school feeding programs, such as, “Programa Nacional de Alimentação Escolar (PNAE)” in Brazil [72], and the “National School Lunch Program (NLSP)” in the United States [74], for example.

### 3.3. Food-School Environment Exposure and Associated Dietary-Nutritional-Health Implications

The association between the food environment around schools and health-related outcomes was examined by 20 studies [2,12,13,14,24,26,40,46,47,49,54,55,56,57,58,59,60,63,64,65], which evaluated the following indicators in children and adolescents: overweight/obesity (*n* = 12), body mass index (BMI) (*n* = 9), score BMI (*n* = 5), BMI percentile (*n* = 7), body fat percentage (*n* = 6), and fat mass index (*n* = 1). Of these, three studies found that schools with the highest number of cafeterias, fast-food restaurants, and food advertisements in the territory had a higher proportion of obese children [2,12,13,46], and the others did not find significant differences. Two reasons can be attributed to this result: (1) the reduced sample of some studies, which are not representative of the population; (2) the use of body mass index (BMI) as an easily measured indicator, but it is not so predictive among adolescents, due to the constant fluctuations in weight and height that are characteristic of this stage of life. Some studies call the environment “obesogenic” when there is little or no availability of healthy foods, an ample supply of ultra-processed foods, and exposure to advertising, in addition to discouraging physical activity [2,12,54,55].

Regarding access to supermarkets and convenience stores and the relationship with overweight/obesity, 30% of the studies that performed this analysis (*n* = 6) found a negative association with childhood obesity. In contrast, the other half found positive or null associations. The lack of findings may be due to the complexity and low validity of tools used to measure eating behaviors. Evidence came mainly from developed countries such as the United States, Canada, and the United Kingdom, especially from large-scale investigations, two of them based on national research projects [2,12,20,47,55].

Despite these data, reaching an assertive conclusion on the association between access to supermarkets and childhood obesity involves complex issues, which may have numerous implicit relationships. The first is methodological: no standardized measures and distances were found between the studies, and different indexes were chosen to evaluate obesity. In addition, concerning supermarkets, grocery stores, and convenience stores, most people tend to frequent the environments closest to their homes, which is why it is not easy to associate specifically with the school environment [47,54,64].

Another complex factor in the analysis is the responsibility for acquiring food in these environments, in most cases, is bought by financially responsible families, who provide access to food (healthy or not) to children and adolescents [24,32,47]. Once the analysis was performed considering only the schools’ surroundings and anthropometric index in 45% (*n* = 9) of the analyzed studies, the quantification of food purchased in supermarkets, convenience stores, and grocery stores could be underestimated, impairing the analyses that determine the associations [2,13,55]. On the other hand, these same environments also sell healthy foods, favoring healthy choices, depending on the eating habits of those who will buy the food. In these places, awareness of the population’s negative outcomes of eating low-nutritional quality foods and constant nutrition education activities is needed, aiming to reach as many individuals of all age groups [12,14,47,54,65].

When considering exposure to the food environment and nutritional and health outcomes, it is also necessary to investigate the causes of food insecurity, which permeate choices (individual and collective), and have strong relationships with eating behavior, cultural, and socioeconomic factors. This point was addressed by 60% (*n* = 12) of the studies, which highlighted the importance of considering a family context in which purchasing power is limited. In general, choices tend to be based on the food’s palatability and satiety, regardless of its nutritional value [24,61,65,73]. A study emphasized that it is often reinforced by the social environment and the influence individuals suffer from the media and food advertising [13].

In the periphery, it is more accessible to shop in grocery markets and small mixed convenience stores, but they hardly offer a variety of healthy options, such as fresh products, whole grains, and lean meats [28,73,75,76,77]. The cost of these foods mentioned is another factor that can make their acquisition difficult, since with the expressive increase in food commercialization values, the choice for those that bring immediate satiety and that can be prepared with less expense tends to be more, increasing consumption of industrialized foods.

Reflecting on food security, especially in the school context, it is important to consider the access that children and adolescents have to food in their homes, in addition to the surroundings of schools themselves. Considering individuality and the social, physical, and economic context in which these individuals live, which undoubtedly influence their eating behavior and are reflected in the choices they will possibly make when purchasing food around the school [16,73,75,76,77,78].

## 4. Strengths, Limitations, and Closing Remarks

This systematic scoping review is the first to focus exclusively on food environment research around schools. The strengths of this review include the use of the PRISMA-ScR guidelines to ensure a robust and repeatable process, the use of six electronic databases to capture the breadth and depth of peer-reviewed publications, the inclusion of quantitative, qualitative, and mixed methods, the use of the conceptual framework to guide reporting and discussion of food environment outcomes for food and nutrition health and security. Regarding the limiting factors of this work, there is the use of secondary data, subject to errors in collection and analysis, and the absence of a standard instrument for assessing the food environment that could support the qualitative assessment of the articles used. Additionally, there is the possibility of outdated information since both the presence of schools and food establishments can have a high turnover and no time cut was done.

This study is subject to methodological limitations due to ecological studies, such as the lack of individual information on behavior and food choices in environments other than the school environment, not considering the variability of the characteristics studied within the groups. In addition, it is difficult to establish a direct cause-and-effect relationship concerning health outcomes since eating in the school environment is not the only variable contributing to future health events. Another limiting aspect was the risk of bias since most studies had moderate risk.

This study demonstrated that the food environment around the schools was characterized by the wide availability of food establishments, primarily, fast-food restaurants, convenience stores, supermarkets, and grocery stores, known for offering a wide variety of unhealthy foods. Identifying a standardized methodology to assess the food environment around the schools was not possible, making it difficult to compare the results presented by the studies. Despite this, it was possible to identify differences related to the urban and rural environments, concerning the predominant race/ethnicity and income in the neighborhoods where the schools were located, and also the age of the students (children or adolescents). The data about health outcomes related to the food trade in the school environment were not conclusive, but they raised the discussion about the need to expand healthy alternatives in the surroundings of schools as well as the performance of food and nutrition education activities, and also of regulatory strategies by the government, given the socioeconomic implications that affect access to food. Thus, conducting standardized and comprehensive studies evaluating food choices in the school environment and their interrelationships is very important to ensure children’s food and nutrition security and minimize adverse health outcomes in the medium and long term.

The analysis of the food environment is part of the strategy to improve school feeding and reduce the risks related to food choices in childhood and adolescence and the adverse health outcomes caused by food in this age group. Public policies must be implemented to establish adequate and healthy food programs in the school environment to ensure food is offered in the quantity and quality necessary for students (avoiding purchasing food around schools). Consequently, it makes the environment healthier and improves the quality of life and health prospects of this population.

## Figures and Tables

**Table 1:** Risk of bias summarized assessment for analytical cross-sectional studies assessed by using MAStARI critical appraisal tools [31]. MAStARI: Meta-analysis of statistics and review instrument.

Authors	Year	Risk of Bias
Austin et al. [32]	2005	Moderate
Frank et al. [33]	2006	Moderate
Kipke et al. [34]	2007	Moderate
Simon et al. [35]	2008	Moderate
Sturm [36]	2008	Moderate
Zenk; Powell [37]	2008	Moderate
Davis; Carpenter [26]	2009	Low
Kestens; Daniel [38]	2010	Moderate
Kwate; Loh [39]	2010	Moderate
Laska et al. [40]	2010	Low
Neckerman et al. [41]	2010	High
Robitaille; Bergeron, Lasnier [42]	2010	High
Tester; Yen; Laraia [43]	2010	High
Nixon; Doud [44]	2011	Moderate
Day; Pearce [11]	2011	Low
Gebauer; Laska [45]	2011	Moderate
Sanchez et al. [46]	2012	High
An; Sturm [47]	2012	Moderate
Black; Day [48]	2012	Moderate
Ellaway et al. [49]	2012	Moderate
Forsyth et al. [50]	2012	Moderate
He et al. [8]	2012	Low
Héroux et al. [51]	2012	Moderate
Leite et al. [52]	2012	Moderate
Seliske et al. [53]	2012	Moderate
Buck et al. [14]	2013	Moderate
Day; Pearce; Pearson [54]	2013	Moderate
Grier; Davis [55]	2013	Moderate
Richmond et al. [56]	2013	High
Smith et al. [24]	2013	Moderate
Engler-Stringer et al. [57]	2014	Moderate
Laxer; Janssen [58]	2014	Moderate
Clark et al. [59]	2014	Moderate
Tang et al. [2]	2014	Low
Cutumisu et al. [60]	2017	Moderate
Fitzpatrick et al. [12]	2017	Moderate
Soltero et al. [61]	2017	Moderate
do Carmo et al. [10]	2018	Moderate
Li et al. [62]	2019	Moderate
Lourenço et al. [63]	2019	Moderate
Rummo et al. [64]	2020	Moderate
Chew; Moran; Barnoya [65]	2020	Moderate
Henriques et al. [66]	2021	Moderate
Saavedra-Garcia et al. [13]	2021	Moderate
Leite et al. [3]	2021	Moderate
Peres et al. [67]	2021	Moderate

**Table 2:** Description of included studies with density and/or proximity analysis units and types of food establishments evaluated.

Authors	Year	Local	Sample Size	Unit of Analysis	Types of Food Establishments Evaluated
Austin et al. [32]	2005	Chicago (U.S.)	1292 schools and 613 food establishments	Density of food establishments on a buffer of 400 and 800 m	Fast-food restaurants
Frank et al. [33]	2006	Atlanta (U.S.)	302 food establishments around schools	Density and proximity of food establishments on a buffer of 400, 800, 1200, 1600, and 2000 m	Restaurants, grocery stores, convenience stores, and fast-food restaurants
Kipke et al. [34]	2007	California (U.S.)	11 schools and 190 food establishments	Density of food establishments on a buffer of 300 and 500 m	Fast-food restaurants, bakeries, ice cream parlors, convenience stores, butchers and fishmongers, grocery stores/supermarkets
Simon et al. [35]	2008	California (U.S.)	1684 schools and 2712 food establishments	Density of food establishments on a buffer of 400 and 800 m on school territory	Fast-food restaurants
Sturm [36]	2008	US	31,622 schools	Density of food establishments on buffers of 400 and 800 m	Restaurants (including fast-food outlets), snack and non-alcoholic beverage stores, convenience stores, liquor distributors, and liquor stores
Zenk; Powell [37]	2008	US	31,243 schools	Density of food establishments on buffers of 800 m	Convenience stores and fast-food restaurants
Davis; Carpenter [26]	2009	California (US)	>500,000 students	Density of food establishments on buffer of 800 m	Fast-food restaurants
Kestens; Daniel [38]	2010	Montreal (Canada)	1168 schools and 7368 food establishments	Density of food establishments on a buffer of 750 m on school territory	Fast-food restaurants, fruit and vegetable stores, and full-service restaurants
Kwate; Loh [39]	2010	New York (U.S.)	2096 schools and 817 food establishments	Density of food establishments on a buffer of 400 m on school territory	Fast-food restaurants
Laska et al. [40]	2010	Minneapolis (U.S.)	349 adolescents	Density of food establishments on a buffer of 800, 1600, and 3000 m	Restaurants, fast-food restaurants, convenience stores, grocery stores, and other food establishments
Neckerman et al. [41]	2010	New York (U.S.)	1089 schools	Density of food establishments on a buffer of 400 and 800 m	Restaurants, fast-food restaurants, convenience stores, grocery stores, and other food establishments
Robitaille; Bergeron, Lasnier [42]	2010	Quebec (Canada)	2302 schools and 5233 food establishments	Density of food establishments on a buffer of 400 and 640 m	Convenience stores and fast-food restaurants
Tester; Yen; Laraia [43]	2010	California (U.S.)	6 schools	Density of food establishments on a buffer of 400 m	Street vendors
Nixon; Doud [44]	2011	California(US)	41 schools	Density of food establishments on a buffer of 400 and 800 m	Fast-food restaurants
Day; Pearce [11]	2011	New Zealand	406 schools 1849 food establishments	Density of food establishments on a buffer of 400 and 800 m	Convenience stores and fast-food restaurants
Gebauer; Laska [45]	2011	Minneapolis (U.S.)	36 schools and 63 food establishments	Density of food establishments on a buffer of 800 m	Convenience stores
Sanchez et al. [46]	2012	California (U.S.)	926,018 children from 6362 schools	Proximity of food establishments to schools	Fast-food restaurants and convenience stores
An; Sturm [47]	2012	California (U.S.)	8226 children and 5236 adolescents	Density and proximity of food establishments on a buffer of 160, 800, 1600, and 2400 m on school territory	Fast-food restaurants, convenience stores, mini-markets, grocery stores, and supermarkets
Black; Day [48]	2012	British Columbia (Canada)	1392 schools	Density of food establishments on a buffer of 800 m	Fast-food restaurants, liquor stores, eateries, delis, and convenience stores
Ellaway et al. [49]	2012	Glasgow (Reino Unido)	29 schools and 2236 food establishments	Density of food establishments on a buffer of 400 and 800 m	Cafes, takeaways (food for off-site consumption), fast-food restaurants, general stores (such as kiosks and supermarkets), trailers
Forsyth et al. [50]	2012	Minneapolis (U.S.)	2724 adolescents in 20 schools	Density of food establishments on a buffer of 800 and 1600 m in school territory	Fast-food restaurants
He et al. [8]	2012	Ontario (Canada)	632 adolescents in 21 schools	Density of food establishments on a buffer of 1 km	Convenience stores and fast-food restaurants
Héroux et al. [51]	2012	Canada, Escócia, and U.S.	26,778 students of 687 schools and 46 food establishments	Density of food establishments on a buffer of 1 km	Convenience stores, coffee shops, and fast-food restaurants
Leite et al. [52]	2012	Santos (Brazil)	3 schools and 82 food establishments	Density of food establishments on a buffer of 500 m	Food establishments classified in the predominant sale of minimally processed and ultra-processed foods
Seliske et al. [53]	2012	Canada	6971 students from 158 schools	Density of food establishments on a buffer of 500, 750, 1000, 1500, 2000, and 5000 m	Convenience stores, fast-food restaurants, and coffee shops
Buck et al. [14]	2013	Delmenhorst (Germany)	384 children and 188 food establishments	Density of food establishments on a buffer of 1.5 km in school territory	Fast-food restaurants, snack bars, kebab shops, bakeries, kiosks, grocery stores, and supermarkets
Day; Pearce; Pearson [54]	2013	Christchurch (New Zealand)	Schools and food establishments from 1966 to 2006	Density of food establishments on a buffer of 800 m	Supermarkets/grocery stores, convenience stores, fast-food restaurants
Grier; Davis [55]	2013	California (U.S.)	Schools	Proximity of food establishments to schools	Fast-food restaurants
Richmond et al. [56]	2013	Massachusetts (U.S.)	18,281 students from 47 schools	Density of food establishments on a buffer of 1.5 km	Convenience stores and fast-food restaurants
Smith et al. [24]	2013	London (England)	757 students from 30 schools	Density of food establishments on a buffer of 400 and 800 m	Grocery stores, convenience stores, and takeaways (food for off-site consumption)
Engler-Stringer et al. [57]	2014	Saskatoon (Canada)	76 schools, 375 food establishments	Density of food establishments on a buffer of 750 m	Grocery stores, convenience stores, and fast-food restaurants
Laxer; Janssen [58]	2014	Canada	6099 adolescents from 255 schools	Density of food establishments on a buffer of 1 km	Fast-food restaurants
Clark et al. [59]	2014	Otago (New Zealand)	730 students from 11 schools	Density of food establishments on a buffer of 800 m and 1500 m in school territory	Supermarkets, grocery stores, convenience stores, fast-food restaurants
Tang et al. [2]	2014	Camden, New Brunswick, Newark e Trenton (New Zealand)	8 schools	Density of food establishments on a buffer of 400 m	Supermarkets, grocery stores, convenience stores, fast-food restaurants
Cutumisu et al. [60]	2017	Quebec (Canada)	374 schools	Density of food establishments on buffer of 750 m	Fast-food restaurants
Fitzpatrick et al. [12]	2017	Quebec (Canada)	246 schools	Density of food establishments on a buffer of 750 m	Convenience stores and fast-food restaurants
Soltero et al. [61]	2017	Guadalajara, Puerto Vallarta, and Mexico City (Mexico)	32 schools	Density of food establishments on a buffer of 800 m	Supermarkets, grocery stores, convenience stores, table service restaurants, fast-food restaurants, street vendors, taco stands
do Carmo et al. [10]	2018	Brazil	1247 schools	Direct observation of food establishments in and around schools	Canteens and street vendors
Li et al. [62]	2019	US	52,375 schools	Density of food establishments on a buffer of 800 m	Supermarkets, grocery stores, convenience stores, restaurants
Lourenço et al. [63]	2019	Brazil	962 children from 4 schools	Direct observation of food establishments in and around schools	Canteens and street vendors
Rummo et al. [64]	2020	New York (US)	361,942 students from 706 schools	Density of food establishments on a buffer of 400 and 800 m	Fast-food restaurants, a la carte restaurants, corner stores, supermarkets
Chew; Moran; Barnoya [65]	2020	Guatemala	60 schools	Density of food establishments on a buffer of 150 m	Fast-food restaurants, corner stores, supermarkets, farmer’s stores
Henriques et al. [66]	2021	Niterói, Brazil	56 schools	Direct observation of establishments in and around schools (up to 500 m)	Formal and informal food trade
Saavedra-Garcia et al. [13]	2021	Lima	15 schools	Direct observation of establishments in and around schools	Canteens and street vendors
Leite et al. [3]	2021	Juiz de Fora (Minas Gerais- Brazil)	316 schools and 4690 food establishments	Density of food establishments on a buffer of 250 m, 500 m, and 1000 m	Establishments that sell only or mainly in natural or minimally processed foods; mixed establishments; establishments that sell only or primarily ultra-processed foods; supermarkets and hypermarkets
Peres et al. [67]	2021	Belo Horizonte (Minas Gerais- Brazil)	1436 schools	Density of food establishments on a buffer of 250 m	Supermarkets, hypermarkets, grocery stores, snack bars, candy stores, bars, restaurants, bakeries

## Data Availability

Not applicable.
